# Supplementary material for: Magnetoencephalography recordings reveal the neural mechanisms of auditory contributions to improved visual detection
Source: Commun Biol. 2023 Jan 6;6:12. doi: 10.1038/s42003-022-04335-3 (PMC9816120; doi:10.1038/s42003-022-04335-3)
Supplement: Supplementary file 2 — Supplementary Information [file 42003_2022_4335_MOESM2_ESM.pdf]

Supplemental Methods:

# *Magnetoencephalography recordings reveal the neural mechanisms of auditory contributions to improved visual detection*

Alexis Pérez-Bellido, Eelke Spaak & Floris P. de Lange

## Experiment 1: Participants hits and false alarms

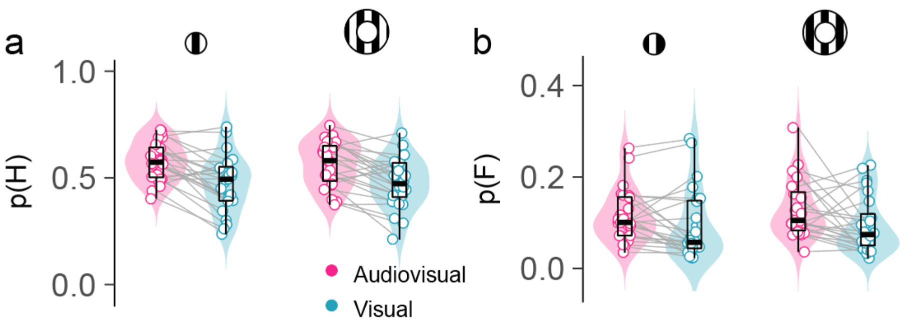


**Supplementary Figure 1** Behavioral results in experiment 1: Proportions of hits (a) and false alarms (b) are depicted for each modality ("audiovisual", "visual"), visual eccentricity condition ("center" and "periphery") and participant. Inside the violin plot, the horizontal black line reflects the median, thick box indicates quartiles, and whiskers 2 ´ the interquartile range. Grey horizontal lines connect participants results across conditions.

## Experiment 1: Control analyses

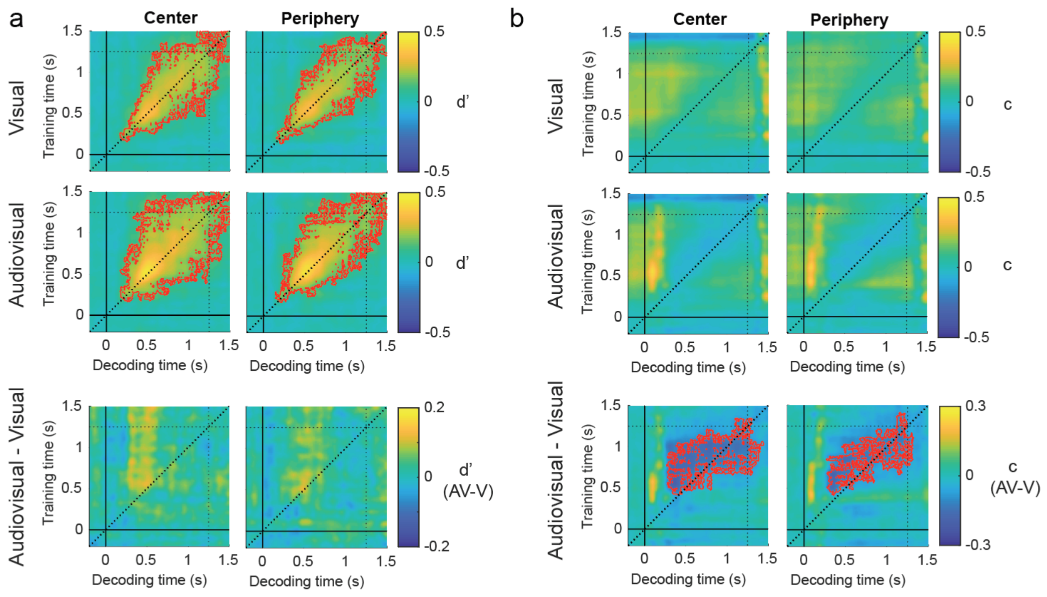


**Supplementary Figure 2** Temporal generalization matrixes depict (a) the neurally decoded sensitivity parameters (d') in the visual (upper TGMs) and audiovisual conditions and their difference, after training and testing the classifiers with data from the center (left panels) and periphery (right panels) visual field conditions (only visual cortex ROI). We found qualitatively similar patterns of enhanced decoding at center and periphery visual fields. We did not find significant clusters, probably due the small number of trials used to train and test the classifiers

but in essence these results demonstrate that the vertical enhanced generalization pattern is present at center and periphery visual fields. b. illustrates similar results as a but using criterion parameters.

## Methods: ROIs definition

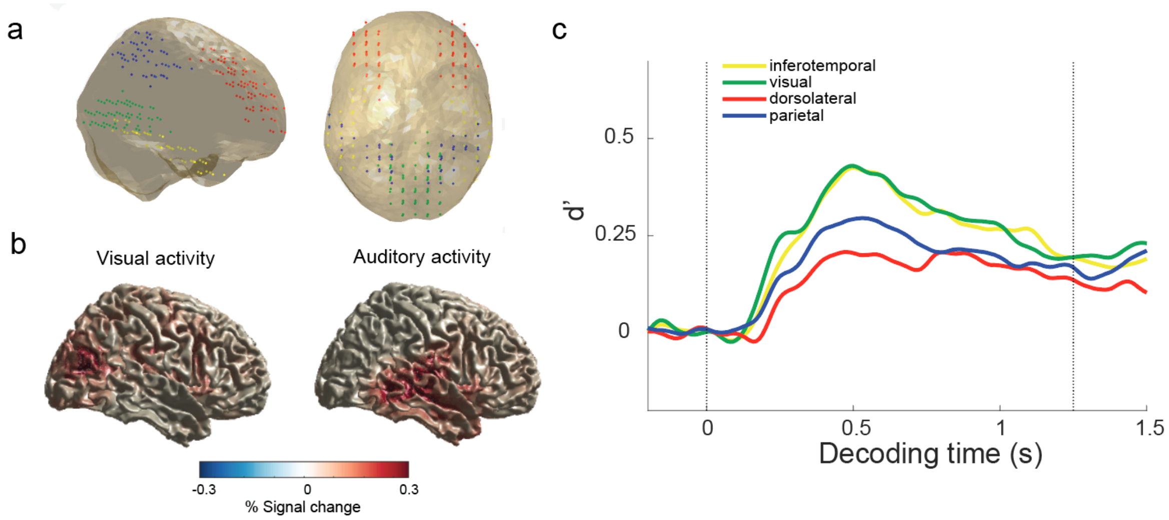


**Supplementary Figure 3** a Grid points conforming each ROI overlayed on two different views of a MNI standard template headmodel (Visual cortex ROI: green points, Inferotemporal cortex: yellow points, Parietal cortex: blue points, Dorsolateral prefrontal cortex: red points). b. We validated that source-reconstructed activity was correctly localized by overlaying group level visual (0.2 to 0.3) and auditory (0.1 to 0.2 s) source-level activation on a surface model of the MNI standard template. The activations were obtained by subtracting S- from S+ trials (visual activity) and A- from A+ trials (auditory activity) in experiment 1. As expected, early visual activations were localized in visual regions (occipital and parietal cortices) and early auditory activations in auditory regions (superior-temporal cortex). c. Decoding sensitivity in experiment 1 (visual and audiovisual modalities together) for each ROI at matching training and decoding time points (diagonal of the TGM): Information decoding peaks at 500 ms and nicely follows the temporal ordering expected for each ROI as a function of its level within the perceptual hierarchy.

## Experiment 1: Neurally decoded SDT parameters

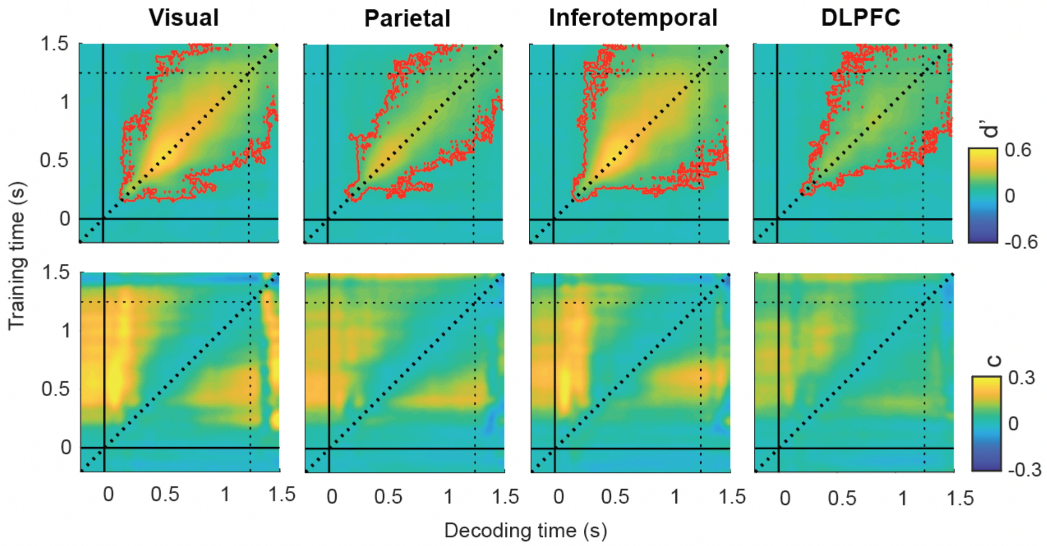


**Supplementary Figure 4** Decoded sensitivity (upper panels) and criterion parameters (lower panels) in each ROI training the classifier in the visual and audiovisual conditions together. Red thin contours depict significant clusters.

## Experiment 1: Temporal ordering of clusters across ROIs

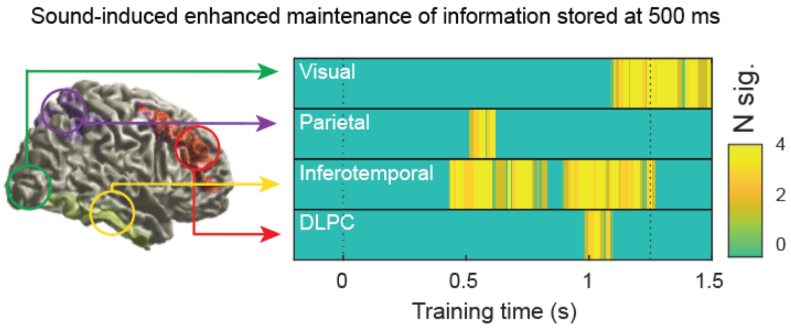


**Supplementary Figure 5** The information encoded at 500 ms can be better decoded in the audiovisual compared to the visual condition at different latencies in each ROI. Here we illustrate the temporal ordering of the significant cluster across the different ROIs. Although these results do not allow to make strong claims about the spatial dynamics of the sound-induced visual enhancement, they can inspire new testable hypotheses. Colorbar represents the total number of significant timepoints within a temporal window of 0.45 to 0.55 s in the "decoding time" axis along the "training time" axis in Figure 4. The two vertical dotted lines represent the stimulus onset and the beginning of the response phases respectively.

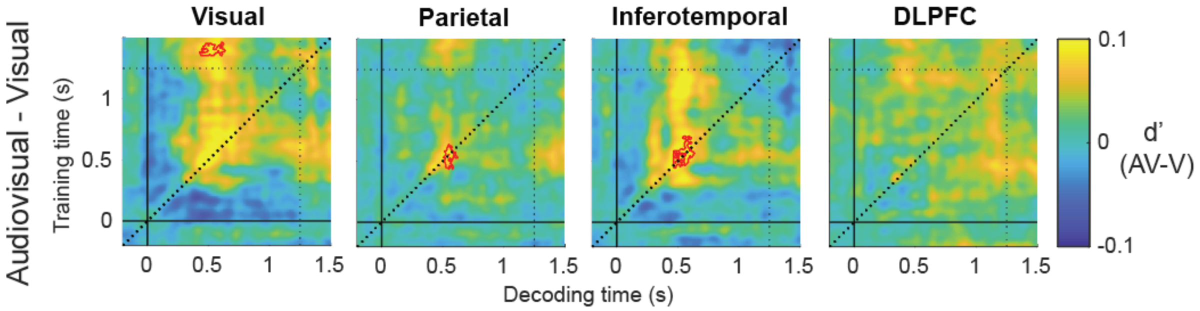


**Supplementary Figure 6** Differential d' temporal generalization matrixes generated by training and testing the decoders in the visual and audiovisual conditions separately. This control analysis recovers the expected symmetry with respect to the diagonal (unity line), that we did not observe when the decoders were trained in both modalities together.

## Decoded hits and false alarms in experiment 1

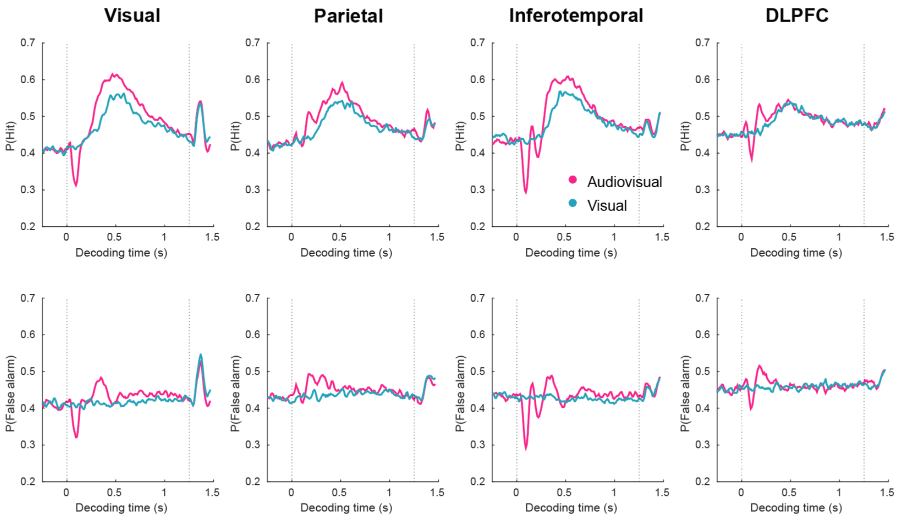


**Supplementary Figure 7** Proportion of hits (upper panels) and false alarms (bottom panels) estimated by a classifier trained at the decoding peak (500 ms) in experiment 1. The visual and audiovisual modalities are represented with green and magenta lines respectively.

## Experiment 2: Decoded sensitivity and criterion parameters in Low contrast condition

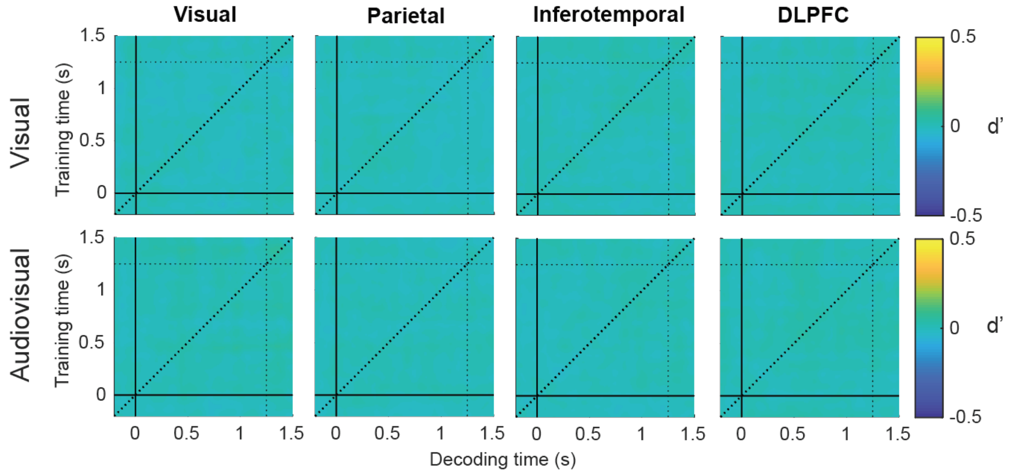


**Supplementary Figure 8** Temporal generalization matrixes depict (A) the neurally decoded sensitivity parameters (d') in the low contrast visual (upper TGMs) and audiovisual (bottom TGMs) conditions for each ROI (Experiment 2).

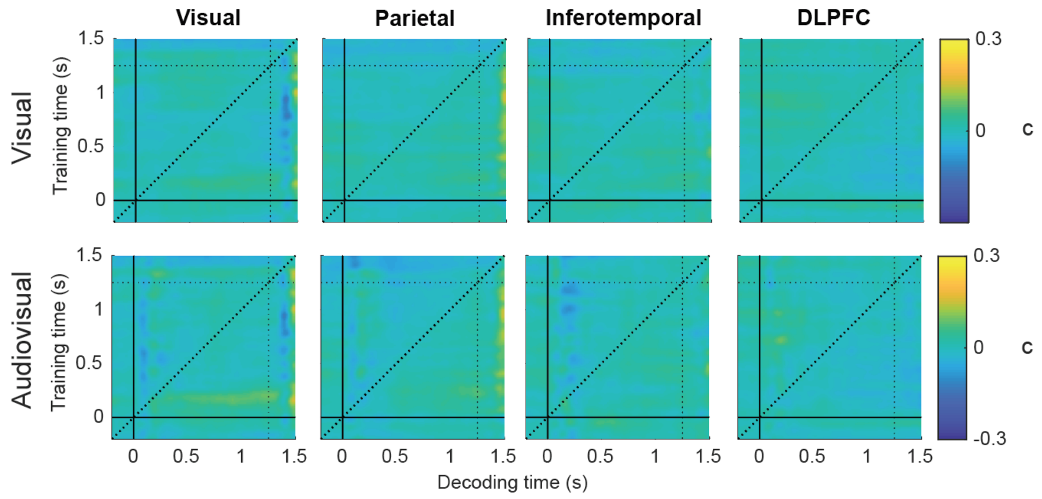


**Supplementary Figure 9** Temporal generalization matrixes depict the neurally decoded criterion parameter in the low contrast visual (upper TGMs) and audiovisual (bottom TGMs) conditions for each ROI (Experiment 2).

## Decoded hits and false alarms in experiment 2

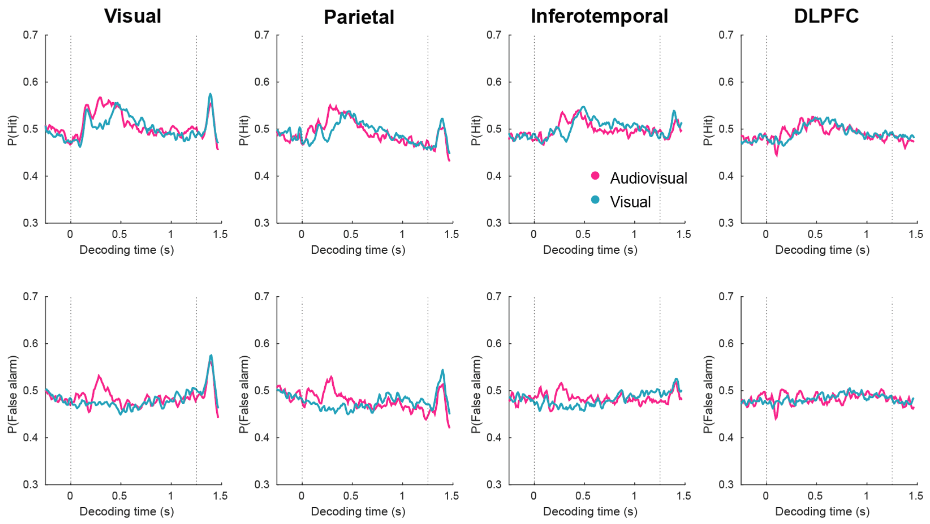


**Supplementary Figure 10** Proportion of hits (upper panels) and false alarms (bottom panels) estimated by a classifier trained at (500 ms) in experiment 2. The visual and audiovisual modalities are represented using green and red lines respectively. See that there is a larger proportion of decoded false alarms at 200 to 350 ms in the audiovisual compared to the visual condition.

## Experiment 2: Univariate modulations in visual cortex ROI

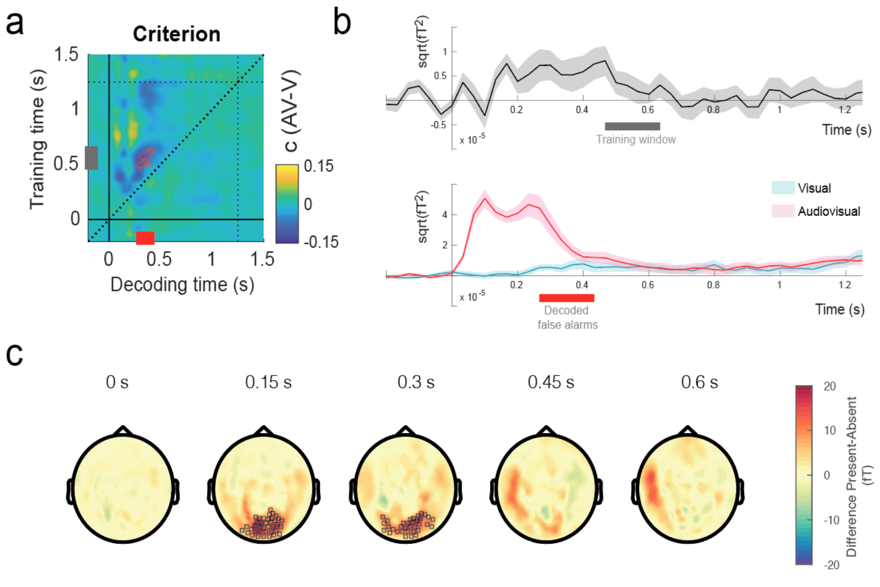


**Supplementary Figure 11** Stimulus-induced univariate modulations as a function of differences in neurally decoded criterion in the visual cortex ROI. a. Temporal generalization matrix showing the cluster containing the

significant difference in criterion between the audiovisual and visual conditions in the visual cortex ROI. Grey and red rectangles depict the temporal windows in which multiple classifiers were trained and tested, leading to a systematic positive bias in signal presence classification (i.e. criterion reduction). b represents the difference between S++ and S- source reconstructed ERF activity in the visual ROI (averaged over virtual channels) regardless of modality (upper panel), and the ERF activity measured in the audiovisual (magenta) and visual (green) conditions (bottom panel). Line contours depict standard error. c Group-level scalp topography for the high contrast condition showing sensor-level S++ against S- ERF differences (averaging over visual and audiovisual conditions). Black boxes represent those MEG sensors that formed part of a significant cluster in each time point.

## Experiment 1 and 2: Univariate modulations across ROIs

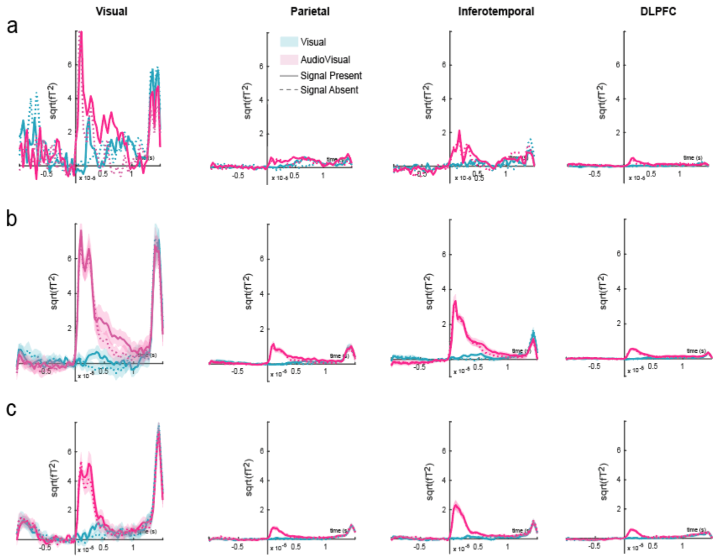


**Supplementary Figure 12** Individual (a) and group average (b and c) of the event-related field potentials (ERFs) projected through the virtual channels conforming each ROI (presented in different columns). The continuous lines represent activity in the S+ trials and the dashed lines represent activity in the S- trials. Line contours depict standard error. Panel a represents source-reconstructed activity for one subject that participated in the first experiment. In panel b and c, group average activity of all the participants in the first experiment and second experiments respectively (in experiment 2, S- and S++ conditions).

## Experiment 1: Neurally decoded SDT parameters and their correlation with behavioral performance

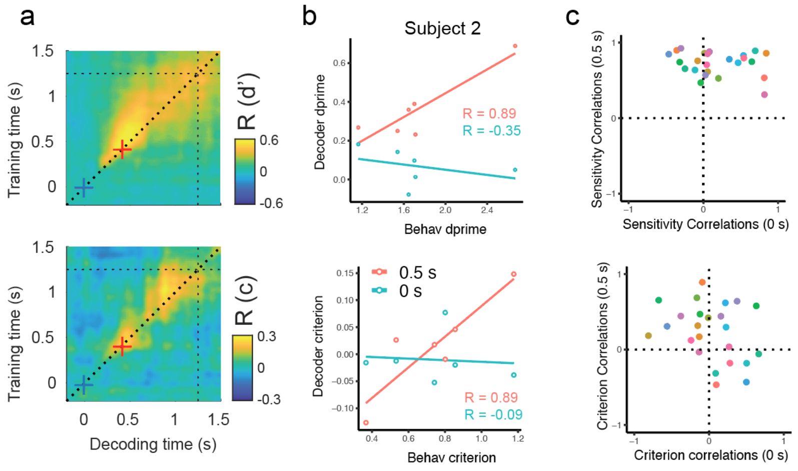


**Supplementary Figure 13** a. Group level temporal generalization matrixes with d' and c correlation values in the first experiment. Blue and red crosses indicate the two time points of interest (0 to 0.5 s) used in the single-subject correlation analysis showed in b. b. Example of d' and c correlation analysis (upper and bottom panels respectively) for one representative subject at two different time points. Whereas the behavioral and decoded d' and c parameter are positively correlated at 0.5 s, the correlation between behavioral and neurally decoded data at time 0 s is not different from 0. Each point represents the SDT parameter estimates calculated for each experimental block (N = 6). c Group level distribution of correlation estimates at time 0 (x axis) and time 0.5 (y axis) for d' and c parameters. Each color point represents one participant.

**Supplementary Table 1**

| | N | AAL label | AAL label |
|---|---|---|---|
| Visual cortex | 67 | Calcarine L and R | Lingual L and R |
| Parietal cortex | 53 | Parietal Superior L and R | Parietal Inferior L and R |
| Interotemporal cortex | 52 | Temporal Inferior L and R | |
| Dorsolateral prefrontal cortex | 79 | Frontal Middle L and R | |

Table listing the number of gridpoints and AAL atlas labels conforming each anatomically defined region of interest.
