# Supplementary material for: Magnetoencephalography recordings reveal the neural mechanisms of auditory contributions to improved visual detection
Source: Commun Biol. 2023 Jan 6;6:12. doi: 10.1038/s42003-022-04335-3 (PMC9816120; doi:10.1038/s42003-022-04335-3)
Supplement: Supplementary file 3 — Description of Additional Supplementary Files [file 42003_2022_4335_MOESM3_ESM.pdf]

Description of Additional Supplementary Files

File name: Supplementary Data 1
Description: Source data underlying Figure 2.

File name: Supplementary Data 2
Description: Source data underlying Figure 7.
