# Supplementary material for: Magnetoencephalography recordings reveal the neural mechanisms of auditory contributions to improved visual detection
Source: Commun Biol. 2023 Jan 6;6:12. doi: 10.1038/s42003-022-04335-3 (PMC9816120; doi:10.1038/s42003-022-04335-3)
Supplement: Supplementary file 6 — Reporting Summary [file 42003_2022_4335_MOESM6_ESM.pdf]

nature portfolio

Corresponding author(s): Alexis Pérez-Bellido

Last updated by author(s): 17/11/22

Reporting Summary

Nature Portfolio wishes to improve the reproducibility of the work that we publish. This form provides structure for consistency and transparency in reporting. For further information on Nature Portfolio policies, see our Editorial Policies and the Editorial Policy Checklist.

Statistics

For all statistical analyses, confirm that the following items are present in the figure legend, table legend, main text, or Methods section.

n/a	Confirmed	
☐	☒	The exact sample size (*n*) for each experimental group/condition, given as a discrete number and unit of measurement
☐	☒	A statement on whether measurements were taken from distinct samples or whether the same sample was measured repeatedly
☐	☒	The statistical test(s) used AND whether they are one- or two-sided<br>*Only common tests should be described solely by name; describe more complex techniques in the Methods section.*
☐	☒	A description of all covariates tested
☐	☒	A description of any assumptions or corrections, such as tests of normality and adjustment for multiple comparisons
☐	☒	A full description of the statistical parameters including central tendency (e.g. means) or other basic estimates (e.g. regression coefficient) AND variation (e.g. standard deviation) or associated estimates of uncertainty (e.g. confidence intervals)
☐	☒	For null hypothesis testing, the test statistic (e.g. *F*, *t*, *r*) with confidence intervals, effect sizes, degrees of freedom and *P* value noted<br>*Give P values as exact values whenever suitable.*
☐	☒	For Bayesian analysis, information on the choice of priors and Markov chain Monte Carlo settings
☒	☐	For hierarchical and complex designs, identification of the appropriate level for tests and full reporting of outcomes
☐	☒	Estimates of effect sizes (e.g. Cohen's *d*, Pearson's *r*), indicating how they were calculated

Our web collection on statistics for biologists contains articles on many of the points above.

Software and code

Policy information about availability of computer code

Data collection	Matlab 15
Data analysis	Matlab 17, Fieldtrip, R

For manuscripts utilizing custom algorithms or software that are central to the research but not yet described in published literature, software must be made available to editors and reviewers. We strongly encourage code deposition in a community repository (e.g. GitHub). See the Nature Portfolio guidelines for submitting code & software for further information.

Data

Policy information about availability of data

All manuscripts must include a data availability statement. This statement should provide the following information, where applicable:

- Accession codes, unique identifiers, or web links for publicly available datasets
- A description of any restrictions on data availability
- For clinical datasets or third party data, please ensure that the statement adheres to our policy

All data and code used for stimulus presentation and analysis are available from the Donders Institute for Brain, Cognition and Behavior repository at https://doi.org/10.34973/2m6r-4167

Human research participants

Policy information about studies involving human research participants and Sex and Gender in Research.

Reporting on sex and gender	Twenty-five healthy human volunteers with normal or corrected-to-normal vision and audition participated in the first (17 females, mean age = 24 years, SD = 6 years) and second experiment (12 females, mean age = 25 years, SD = 7 years). Participants gender was based on their own reports and matched their biological sex. Written informed consent was obtained from each individual prior to the beginning of the experiment. We did not perform sex/gender based analyses given that: 1) We lack of statistical power to find gender related effects. 2) We are interested in investigating neural mechanisms that are independent of participants sex/gender.
Population characteristics	See above.
Recruitment	Participants sign in voluntarily to participate in the experiment through an Internet portal: SONA (https://radboud.sona-systems.com/default.aspx). Participants received either monetary compensation or study credits.
Ethics oversight	The study was approved by the local ethics committee (CMO Arnhem-Nijmegen, Radboud University Medical Center) under the general ethics approval (“Imaging Human Cognition”, CMO 2014/288), and the experiment was conducted in compliance with these guidelines.

Note that full information on the approval of the study protocol must also be provided in the manuscript.

Field-specific reporting

Please select the one below that is the best fit for your research. If you are not sure, read the appropriate sections before making your selection.

☐ Life sciences ☒ Behavioural & social sciences ☐ Ecological, evolutionary & environmental sciences

For a reference copy of the document with all sections, see nature.com/documents/nr-reporting-summary-flat.pdf

Behavioural & social sciences study design

All studies must disclose on these points even when the disclosure is negative.

Study description	In this study we acquired behavioral and neural activity QUANTITATIVE data of human participants while they performed a visual detection task.
Research sample	Radboud University students and Nijmegen citizens: Twenty-five healthy human volunteers with normal or corrected-to-normal vision and audition participated in the first (17 females, mean age = 24 years, SD = 6 years) and second experiment (12 females, mean age = 25 years, SD = 7 years). The sample is representative of the population.
Sampling strategy	The sampling was random but given the characteristics of the task (audiovisual detection), we only selected participants with normal audition and normal or corrected to normal vision. The sample size (N =24) was determined prior to data collection in consonance with previous similar studies, and ensured 80% power to detect medium-to-large effects (Cohen’s d > 0.6).
Data collection	Behavioral data consisted on button presses and were collected using a dedicated computer. We also collected eye-movement data using Eye-Link 1000 in another specially dedicated computer. Whole-brain neural recordings were registered using a 275-channel MEG system with axial gradiometers (CTF MEG Systems, VSM MedTech Ltd.) located in a magnetically shielded room. The neural data were recorded in another computer. During the recordings, only the participant and the main researcher were present, but both stayed in two separated rooms. The researcher was not blind to the experimental conditions but could not interact with the participants responses or affect the conditions ordering during the experiment.
Timing	The first experiment dataset was collected between 03/10/2017 to 09/11/2017. The second experiment dataset was collected between 26/06/2018 to 24/10/2018 (summer break in the middle).
Data exclusions	One subject in the first and one subject in the second experiment were excluded during the preprocessing due to insufficient data quality (severe eye and muscle artifacts and poor performance).
Non-participation	No participants declined participation.
Randomization	We used a repeated measures design in which participants were not allocated to different experimental groups/conditions.

Reporting for specific materials, systems and methods

We require information from authors about some types of materials, experimental systems and methods used in many studies. Here, indicate whether each material, system or method listed is relevant to your study. If you are not sure if a list item applies to your research, read the appropriate section before selecting a response.

Materials & experimental systems

n/a	Involved in the study
☒	☐ Antibodies
☒	☐ Eukaryotic cell lines
☒	☐ Palaeontology and archaeology
☒	☐ Animals and other organisms
☒	☐ Clinical data
☒	☐ Dual use research of concern

Methods

n/a	Involved in the study
☒	☐ ChIP-seq
☒	☐ Flow cytometry
☒	☐ MRI-based neuroimaging
